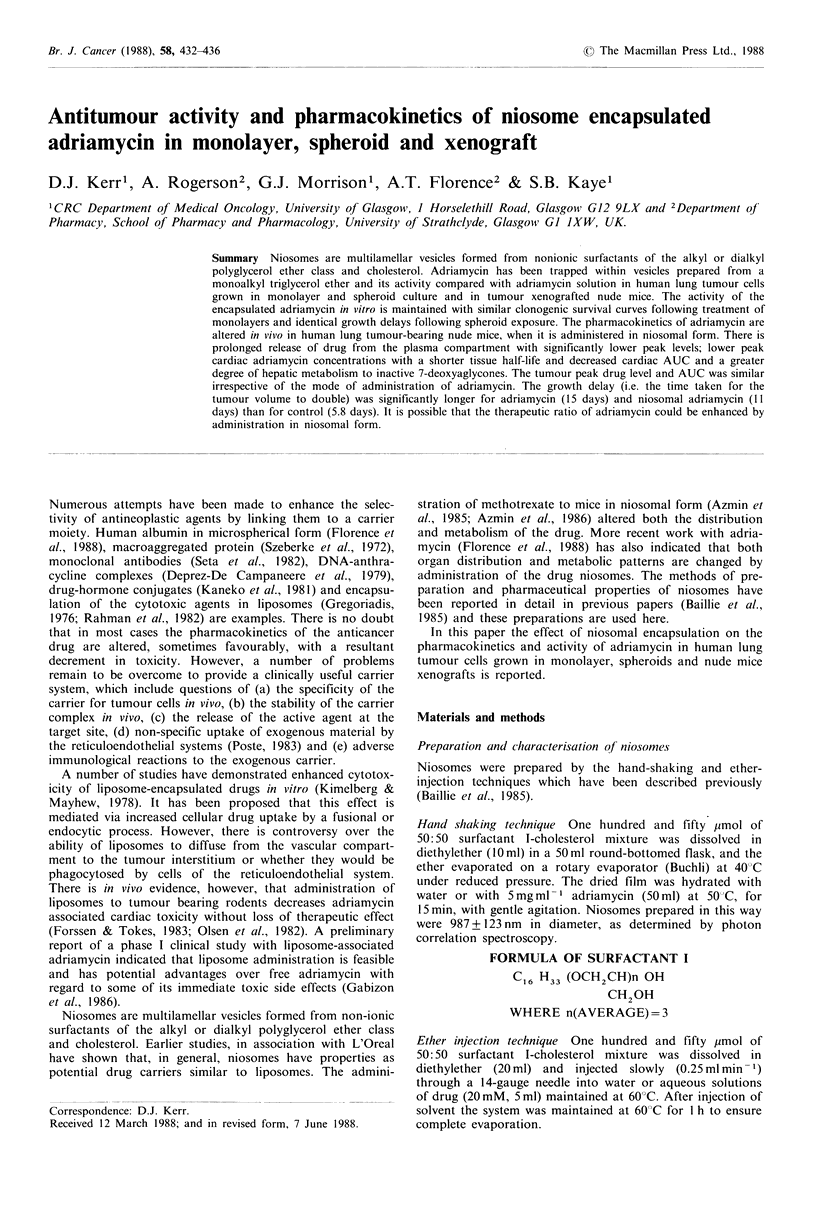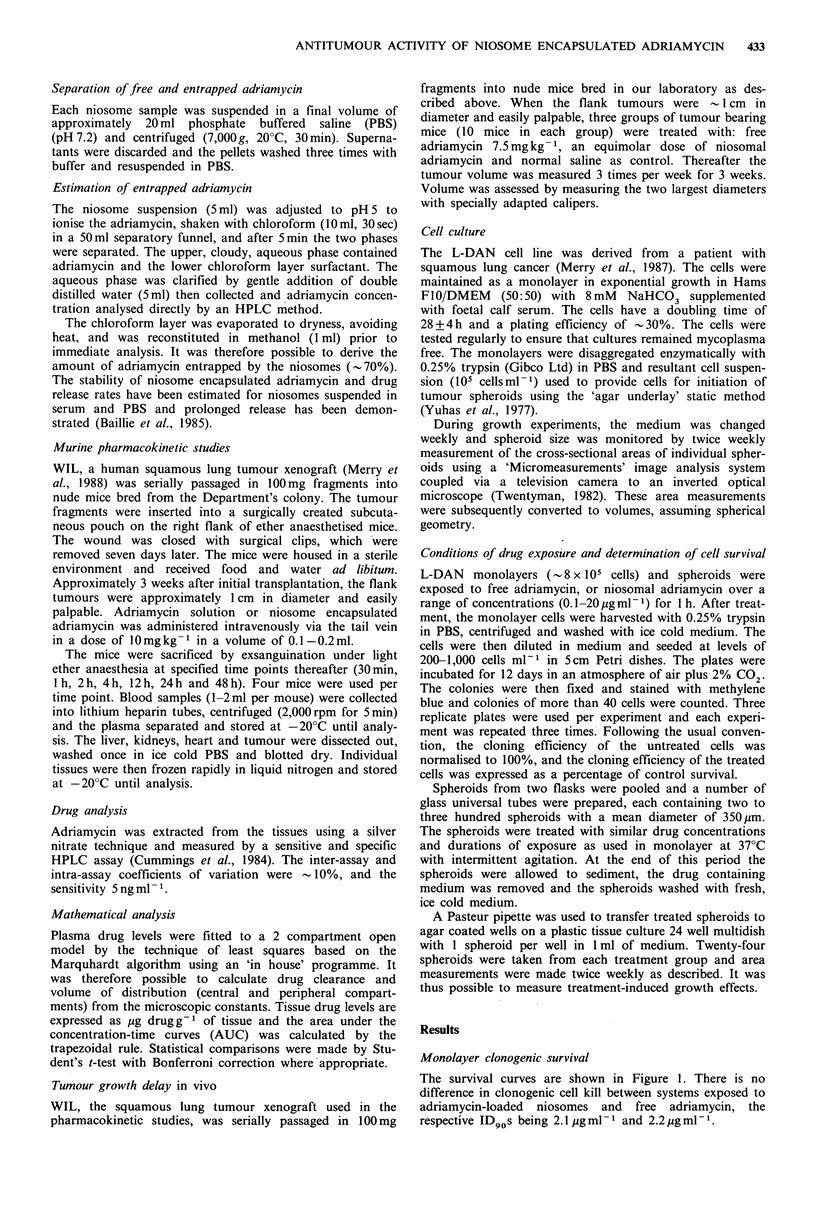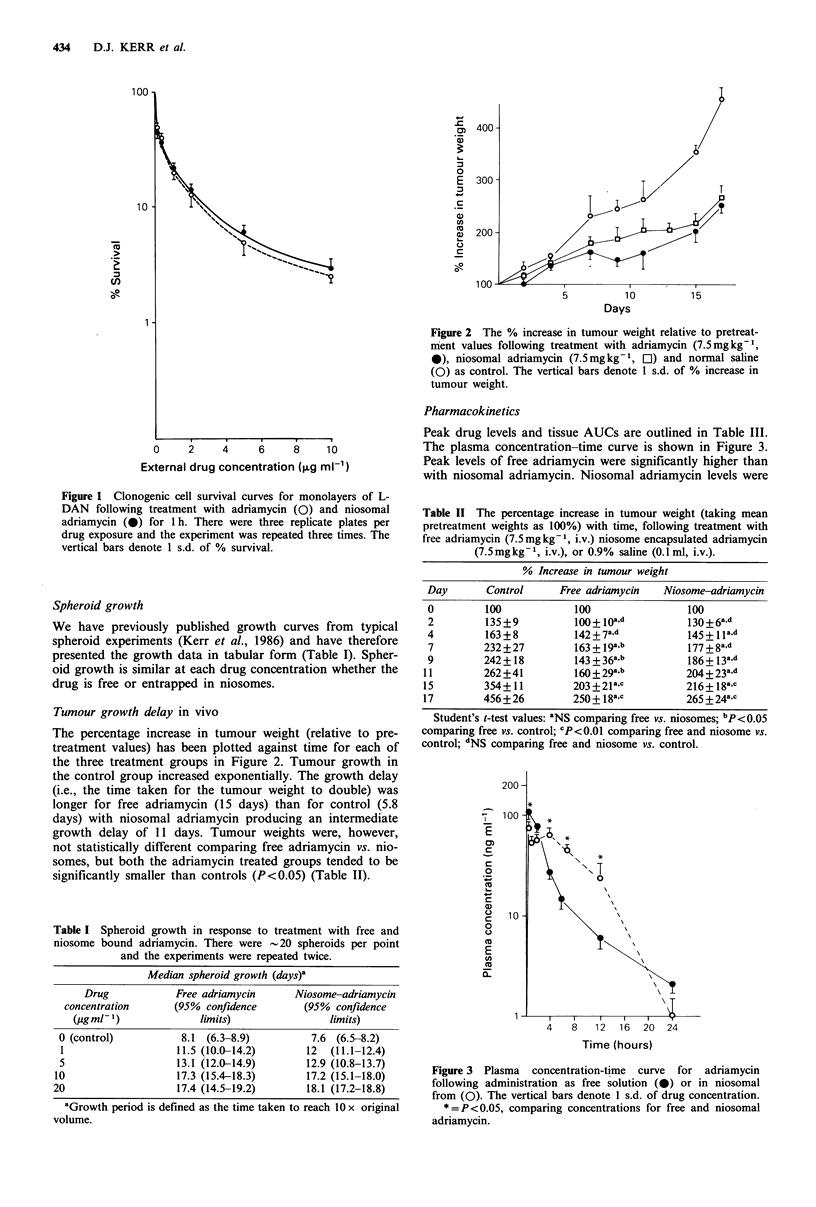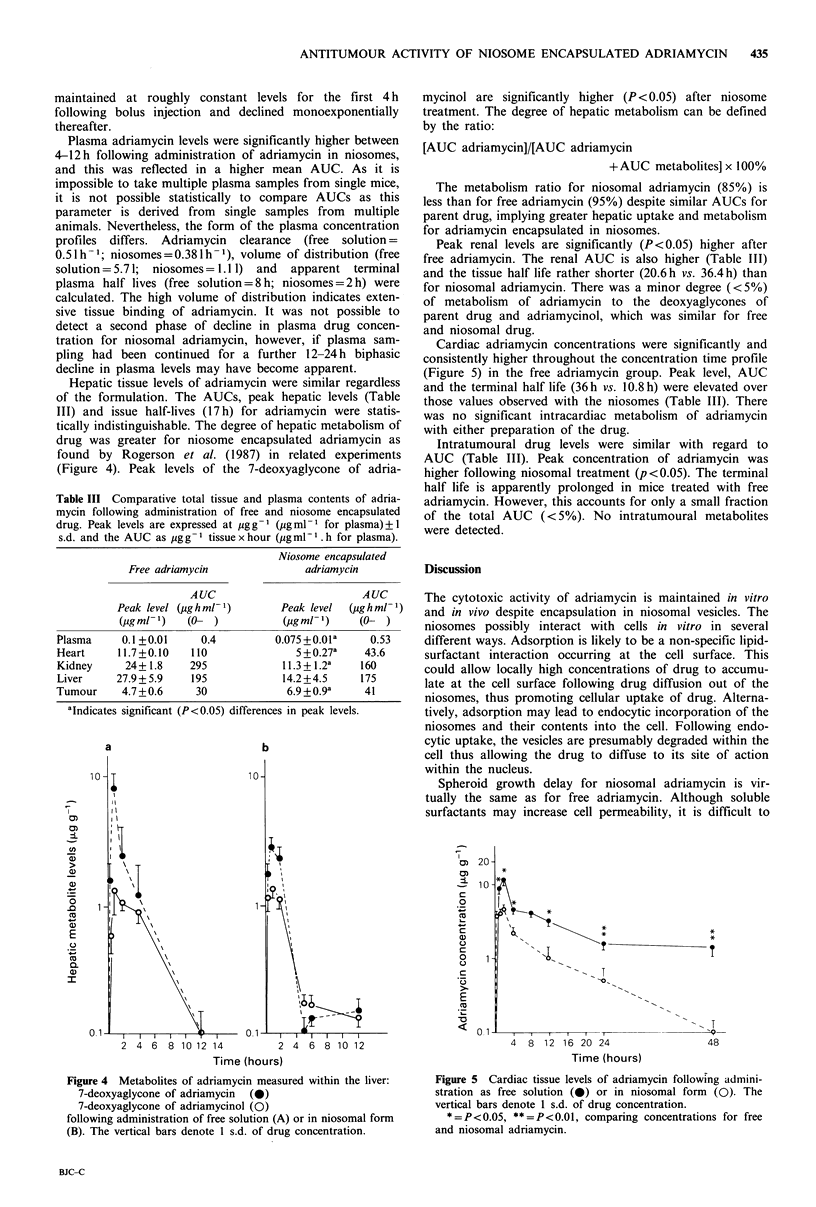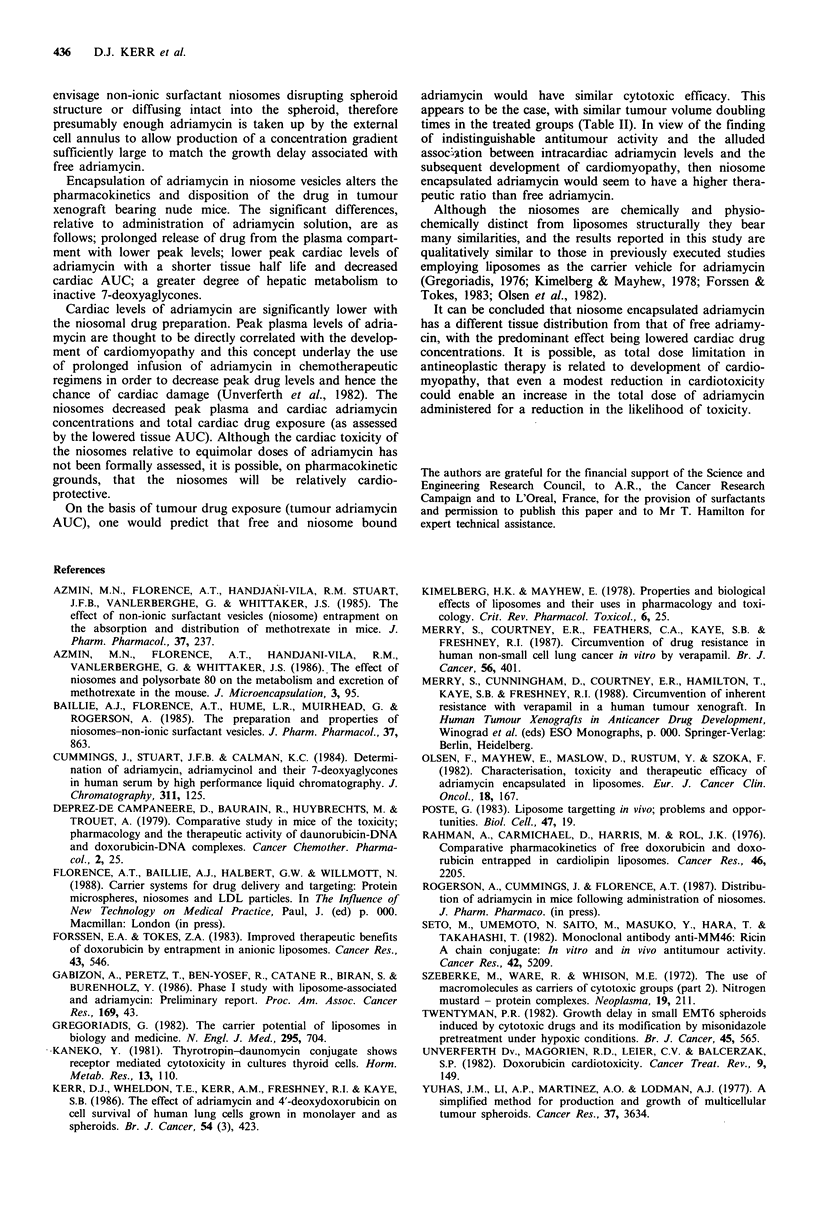# Antitumour activity and pharmacokinetics of niosome encapsulated adriamycin in monolayer, spheroid and xenograft.

**DOI:** 10.1038/bjc.1988.235

**Published:** 1988-10

**Authors:** D. J. Kerr, A. Rogerson, G. J. Morrison, A. T. Florence, S. B. Kaye

**Affiliations:** CRC Department of Medical Oncology, University of Glasgow, UK.

## Abstract

Niosomes are multilamellar vesicles formed from nonionic surfactants of the alkyl or dialkyl polyglycerol ether class and cholesterol. Adriamycin has been trapped within vesicles prepared from a monoalkyl triglycerol ether and its activity compared with adriamycin solution in human lung tumour cells grown in monolayer and spheroid culture and in tumour xenografted nude mice. The activity of the encapsulated adriamycin in vitro is maintained with similar clonogenic survival curves following treatment of monolayers and identical growth delays following spheroid exposure. The pharmacokinetics of adriamycin are altered in vivo in human lung tumour-bearing nude mice, when it is administered in niosomal form. There is prolonged release of drug from the plasma compartment with significantly lower peak levels; lower peak cardiac adriamycin concentrations with a shorter tissue half-life and decreased cardiac AUC and a greater degree of hepatic metabolism to inactive 7-deoxyaglycones. The tumour peak drug level and AUC was similar irrespective of the mode of administration of adriamycin. The growth delay (i.e. the time taken for the tumour volume to double) was significantly longer for adriamycin (15 days) and niosomal adriamycin (11 days) than for control (5.8 days). It is possible that the therapeutic ratio of adriamycin could be enhanced by administration in niosomal form.


*Br. J. Cancer* (1988), **58**, 432–436 © The Macmillan Press Ltd., 1988

### Antitumour activity and pharmacokinetics of niosome encapsulated adriamycin in monolayer, spheroid and xenograft

D.J. Kerr[1], A. Rogerson[2], G.J. Morrison[1], A.T. Florence[2] & S.B. Kaye[1]

[1]*CRC Department of Medical Oncology, University of Glasgow, 1 Horselethill Road, Glasgow G12 9LX and* [2]*Department of Pharmacy, School of Pharmacy and Pharmacology, University of Strathclyde, Glasgow G1 1XW, UK.*

**Summary** Niosomes are multilamellar vesicles formed from nonionic surfactants of the alkyl or dialkyl polyglycerol ether class and cholesterol. Adriamycin has been trapped within vesicles prepared from a monoalkyl triglycerol ether and its activity compared with adriamycin solution in human lung tumour cells grown in monolayer and spheroid culture and in tumour xenografted nude mice. The activity of the encapsulated adriamycin *in vitro* is maintained with similar clonogenic survival curves following treatment of monolayers and identical growth delays following spheroid exposure. The pharmacokinetics of adriamycin are altered *in vivo* in human lung tumour-bearing nude mice, when it is administered in niosomal form. There is prolonged release of drug from the plasma compartment with significantly lower peak levels; lower peak cardiac adriamycin concentrations with a shorter tissue half-life and decreased cardiac AUC and a greater degree of hepatic metabolism to inactive 7-deoxyaglycones. The tumour peak drug level and AUC was similar irrespective of the mode of administration of adriamycin. The growth delay (i.e. the time taken for the tumour volume to double) was significantly longer for adriamycin (15 days) and niosomal adriamycin (11 days) than for control (5.8 days). It is possible that the therapeutic ratio of adriamycin could be enhanced by administration in niosomal form.

Numerous attempts have been made to enhance the selectivity of antineoplastic agents by linking them to a carrier moiety. Human albumin in microspherical form (Florence *et al.*, 1988), macroaggregated protein (Szeberke *et al.*, 1972), monoclonal antibodies (Seta *et al.*, 1982), DNA-anthracycline complexes (Deprez-De Campaneere *et al.*, 1979), drug-hormone conjugates (Kaneko *et al.*, 1981) and encapsulation of the cytotoxic agents in liposomes (Gregoriadis, 1976; Rahman *et al.*, 1982) are examples. There is no doubt that in most cases the pharmacokinetics of the anticancer drug are altered, sometimes favourably, with a resultant decrement in toxicity. However, a number of problems remain to be overcome to provide a clinically useful carrier system, which include questions of (a) the specificity of the carrier for tumour cells *in vivo*, (b) the stability of the carrier complex *in vivo*, (c) the release of the active agent at the target site, (d) non-specific uptake of exogenous material by the reticuloendothelial systems (Poste, 1983) and (e) adverse immunological reactions to the exogenous carrier.

A number of studies have demonstrated enhanced cytotoxicity of liposome-encapsulated drugs *in vitro* (Kimelberg & Mayhew, 1978). It has been proposed that this effect is mediated via increased cellular drug uptake by a fusional or endocytic process. However, there is controversy over the ability of liposomes to diffuse from the vascular compartment to the tumour interstitium or whether they would be phagocytosed by cells of the reticuloendothelial system. There is *in vivo* evidence, however, that administration of liposomes to tumour bearing rodents decreases adriamycin associated cardiac toxicity without loss of therapeutic effect (Forssen & Tokes, 1983; Olsen *et al.*, 1982). A preliminary report of a phase I clinical study with liposome-associated adriamycin indicated that liposome administration is feasible and has potential advantages over free adriamycin with regard to some of its immediate toxic side effects (Gabizon *et al.*, 1986).

Niosomes are multilamellar vesicles formed from non-ionic surfactants of the alkyl or dialkyl polyglycerol ether class and cholesterol. Earlier studies, in association with L'Oreal have shown that, in general, niosomes have properties as potential drug carriers similar to liposomes. The administration of methotrexate to mice in niosomal form (Azmin *et al.*, 1985; Azmin *et al.*, 1986) altered both the distribution and metabolism of the drug. More recent work with adriamycin (Florence *et al.*, 1988) has also indicated that both organ distribution and metabolic patterns are changed by administration of the drug niosomes. The methods of preparation and pharmaceutical properties of niosomes have been reported in detail in previous papers (Baillie *et al.*, 1985) and these preparations are used here.

In this paper the effect of niosomal encapsulation on the pharmacokinetics and activity of adriamycin in human lung tumour cells grown in monolayer, spheroids and nude mice xenografts is reported.

### Materials and methods

### *Preparation and characterisation of niosomes*

Niosomes were prepared by the hand-shaking and ether-injection techniques which have been described previously (Baillie *et al.*, 1985).

*Hand shaking technique* One hundred and fifty μmol of 50:50 surfactant I-cholesterol mixture was dissolved in diethylether (10 ml) in a 50 ml round-bottomed flask, and the ether evaporated on a rotary evaporator (Buchli) at 40°C under reduced pressure. The dried film was hydrated with water or with 5 mg ml$^{-1}$ adriamycin (50 ml) at 50°C, for 15 min, with gentle agitation. Niosomes prepared in this way were 987 ± 123 nm in diameter, as determined by photon correlation spectroscopy.

**FORMULA OF SURFACTANT I**

$$C_{16}H_{33}(OCH_2\underset{\displaystyle CH_2OH}{CH})_nOH$$

WHERE n(AVERAGE) = 3

*Ether injection technique* One hundred and fifty μmol of 50:50 surfactant I-cholesterol mixture was dissolved in diethylether (20 ml) and injected slowly (0.25 ml min$^{-1}$) through a 14-gauge needle into water or aqueous solutions of drug (20 mM, 5 ml) maintained at 60°C. After injection of solvent the system was maintained at 60°C for 1 h to ensure complete evaporation.

Correspondence: D.J. Kerr.
Received 12 March 1988; and in revised form, 7 June 1988.

*Separation of free and entrapped adriamycin*

Each niosome sample was suspended in a final volume of approximately 20 ml phosphate buffered saline (PBS) (pH 7.2) and centrifuged (7,000 *g*, 20°C, 30 min). Supernatants were discarded and the pellets washed three times with buffer and resuspended in PBS.

*Estimation of entrapped adriamycin*

The niosome suspension (5 ml) was adjusted to pH 5 to ionise the adriamycin, shaken with chloroform (10 ml, 30 sec) in a 50 ml separatory funnel, and after 5 min the two phases were separated. The upper, cloudy, aqueous phase contained adriamycin and the lower chloroform layer surfactant. The aqueous phase was clarified by gentle addition of double distilled water (5 ml) then collected and adriamycin concentration analysed directly by an HPLC method.

The chloroform layer was evaporated to dryness, avoiding heat, and was reconstituted in methanol (1 ml) prior to immediate analysis. It was therefore possible to derive the amount of adriamycin entrapped by the niosomes (~70%). The stability of niosome encapsulated adriamycin and drug release rates have been estimated for niosomes suspended in serum and PBS and prolonged release has been demonstrated (Baillie *et al.*, 1985).

*Murine pharmacokinetic studies*

WIL, a human squamous lung tumour xenograft (Merry *et al.*, 1988) was serially passaged in 100 mg fragments into nude mice bred from the Department's colony. The tumour fragments were inserted into a surgically created subcutaneous pouch on the right flank of ether anaesthetised mice. The wound was closed with surgical clips, which were removed seven days later. The mice were housed in a sterile environment and received food and water *ad libitum*. Approximately 3 weeks after initial transplantation, the flank tumours were approximately 1 cm in diameter and easily palpable. Adriamycin solution or niosome encapsulated adriamycin was administered intravenously via the tail vein in a dose of $10\,mg\,kg^{-1}$ in a volume of 0.1 – 0.2 ml.

The mice were sacrificed by exsanguination under light ether anaesthesia at specified time points thereafter (30 min, 1 h, 2 h, 4 h, 12 h, 24 h and 48 h). Four mice were used per time point. Blood samples (1–2 ml per mouse) were collected into lithium heparin tubes, centrifuged (2,000 rpm for 5 min) and the plasma separated and stored at −20°C until analysis. The liver, kidneys, heart and tumour were dissected out, washed once in ice cold PBS and blotted dry. Individual tissues were then frozen rapidly in liquid nitrogen and stored at −20°C until analysis.

*Drug analysis*

Adriamycin was extracted from the tissues using a silver nitrate technique and measured by a sensitive and specific HPLC assay (Cummings *et al.*, 1984). The inter-assay and intra-assay coefficients of variation were ~10%, and the sensitivity $5\,ng\,ml^{-1}$.

*Mathematical analysis*

Plasma drug levels were fitted to a 2 compartment open model by the technique of least squares based on the Marquhardt algorithm using an 'in house' programme. It was therefore possible to calculate drug clearance and volume of distribution (central and peripheral compartments) from the microscopic constants. Tissue drug levels are expressed as $\mu g$ drug $g^{-1}$ of tissue and the area under the concentration-time curves (AUC) was calculated by the trapezoidal rule. Statistical comparisons were made by Student's *t*-test with Bonferroni correction where appropriate.

*Tumour growth delay* in vivo

WIL, the squamous lung tumour xenograft used in the pharmacokinetic studies, was serially passaged in 100 mg fragments into nude mice bred in our laboratory as described above. When the flank tumours were ~1 cm in diameter and easily palpable, three groups of tumour bearing mice (10 mice in each group) were treated with: free adriamycin $7.5\,mg\,kg^{-1}$, an equimolar dose of niosomal adriamycin and normal saline as control. Thereafter the tumour volume was measured 3 times per week for 3 weeks. Volume was assessed by measuring the two largest diameters with specially adapted calipers.

*Cell culture*

The L-DAN cell line was derived from a patient with squamous lung cancer (Merry *et al.*, 1987). The cells were maintained as a monolayer in exponential growth in Hams F10/DMEM (50:50) with 8 mM $NaHCO_3$ supplemented with foetal calf serum. The cells have a doubling time of $28 \pm 4$ h and a plating efficiency of ~30%. The cells were tested regularly to ensure that cultures remained mycoplasma free. The monolayers were disaggregated enzymatically with 0.25% trypsin (Gibco Ltd) in PBS and resultant cell suspension ($10^5$ cells $ml^{-1}$) used to provide cells for initiation of tumour spheroids using the 'agar underlay' static method (Yuhas *et al.*, 1977).

During growth experiments, the medium was changed weekly and spheroid size was monitored by twice weekly measurement of the cross-sectional areas of individual spheroids using a 'Micromeasurements' image analysis system coupled via a television camera to an inverted optical microscope (Twentyman, 1982). These area measurements were subsequently converted to volumes, assuming spherical geometry.

*Conditions of drug exposure and determination of cell survival*

L-DAN monolayers (~$8 \times 10^5$ cells) and spheroids were exposed to free adriamycin, or niosomal adriamycin over a range of concentrations (0.1–20 $\mu g\,ml^{-1}$) for 1 h. After treatment, the monolayer cells were harvested with 0.25% trypsin in PBS, centrifuged and washed with ice cold medium. The cells were then diluted in medium and seeded at levels of 200–1,000 cells $ml^{-1}$ in 5 cm Petri dishes. The plates were incubated for 12 days in an atmosphere of air plus 2% $CO_2$. The colonies were then fixed and stained with methylene blue and colonies of more than 40 cells were counted. Three replicate plates were used per experiment and each experiment was repeated three times. Following the usual convention, the cloning efficiency of the untreated cells was normalised to 100%, and the cloning efficiency of the treated cells was expressed as a percentage of control survival.

Spheroids from two flasks were pooled and a number of glass universal tubes were prepared, each containing two to three hundred spheroids with a mean diameter of 350 $\mu m$. The spheroids were treated with similar drug concentrations and durations of exposure as used in monolayer at 37°C with intermittent agitation. At the end of this period the spheroids were allowed to sediment, the drug containing medium was removed and the spheroids washed with fresh, ice cold medium.

A Pasteur pipette was used to transfer treated spheroids to agar coated wells on a plastic tissue culture 24 well multidish with 1 spheroid per well in 1 ml of medium. Twenty-four spheroids were taken from each treatment group and area measurements were made twice weekly as described. It was thus possible to measure treatment-induced growth effects.

### Results

*Monolayer clonogenic survival*

The survival curves are shown in Figure 1. There is no difference in clonogenic cell kill between systems exposed to adriamycin-loaded niosomes and free adriamycin, the respective $ID_{90}$s being 2.1 $\mu g\,ml^{-1}$ and 2.2 $\mu g\,ml^{-1}$.

**Figure 1** Clonogenic cell survival curves for monolayers of L-DAN following treatment with adriamycin (○) and niosomal adriamycin (●) for 1 h. There were three replicate plates per drug exposure and the experiment was repeated three times. The vertical bars denote 1 s.d. of % survival.

**Figure 2** The % increase in tumour weight relative to pretreatment values following treatment with adriamycin ($7.5 mg kg^{-1}$, ●), niosomal adriamycin ($7.5 mg kg^{-1}$, □) and normal saline (○) as control. The vertical bars denote 1 s.d. of % increase in tumour weight.

### *Spheroid growth*

We have previously published growth curves from typical spheroid experiments (Kerr *et al.*, 1986) and have therefore presented the growth data in tabular form (Table I). Spheroid growth is similar at each drug concentration whether the drug is free or entrapped in niosomes.

### *Tumour growth delay* in vivo

The percentage increase in tumour weight (relative to pretreatment values) has been plotted against time for each of the three treatment groups in Figure 2. Tumour growth in the control group increased exponentially. The growth delay (i.e., the time taken for the tumour weight to double) was longer for free adriamycin (15 days) than for control (5.8 days) with niosomal adriamycin producing an intermediate growth delay of 11 days. Tumour weights were, however, not statistically different comparing free adriamycin *vs.* niosomes, but both the adriamycin treated groups tended to be significantly smaller than controls ($P<0.05$) (Table II).

**Table I** Spheroid growth in response to treatment with free and niosome bound adriamycin. There were ~20 spheroids per point and the experiments were repeated twice.

| | *Median spheroid growth (days)*[a] | |
|---|---|---|
| *Drug concentration* ($\mu g\, ml^{-1}$) | *Free adriamycin (95% confidence limits)* | *Niosome–adriamycin (95% confidence limits)* |
| 0 (control) | 8.1 (6.3–8.9) | 7.6 (6.5–8.2) |
| 1 | 11.5 (10.0–14.2) | 12 (11.1–12.4) |
| 5 | 13.1 (12.0–14.9) | 12.9 (10.8–13.7) |
| 10 | 17.3 (15.4–18.3) | 17.2 (15.1–18.0) |
| 20 | 17.4 (14.5–19.2) | 18.1 (17.2–18.8) |

[a]Growth period is defined as the time taken to reach 10× original volume.

### *Pharmacokinetics*

Peak drug levels and tissue AUCs are outlined in Table III. The plasma concentration–time curve is shown in Figure 3. Peak levels of free adriamycin were significantly higher than with niosomal adriamycin. Niosomal adriamycin levels were

**Table II** The percentage increase in tumour weight (taking mean pretreatment weights as 100%) with time, following treatment with free adriamycin ($7.5 mg kg^{-1}$, i.v.) niosome encapsulated adriamycin ($7.5 mg kg^{-1}$, i.v.), or 0.9% saline (0.1 ml, i.v.).

| | *% Increase in tumour weight* | | |
|---|---|---|---|
| *Day* | *Control* | *Free adriamycin* | *Niosome–adriamycin* |
| 0 | 100 | 100 | 100 |
| 2 | $135\pm9$ | $100\pm10^{a,d}$ | $130\pm6^{a,d}$ |
| 4 | $163\pm8$ | $142\pm7^{a,d}$ | $145\pm11^{a,d}$ |
| 7 | $232\pm27$ | $163\pm19^{a,b}$ | $177\pm8^{a,d}$ |
| 9 | $242\pm18$ | $143\pm36^{a,b}$ | $186\pm13^{a,d}$ |
| 11 | $262\pm41$ | $160\pm29^{a,b}$ | $204\pm23^{a,d}$ |
| 15 | $354\pm11$ | $203\pm21^{a,c}$ | $216\pm18^{a,c}$ |
| 17 | $456\pm26$ | $250\pm18^{a,c}$ | $265\pm24^{a,c}$ |

Student's *t*-test values: [a]NS comparing free *vs.* niosomes; [b]$P<0.05$ comparing free *vs.* control; [c]$P<0.01$ comparing free and niosome *vs.* control; [d]NS comparing free and niosome *vs.* control.

**Figure 3** Plasma concentration-time curve for adriamycin following administration as free solution (●) or in niosomal from (○). The vertical bars denote 1 s.d. of drug concentration. * = $P<0.05$, comparing concentrations for free and niosomal adriamycin.

maintained at roughly constant levels for the first 4h following bolus injection and declined monoexponentially thereafter.

Plasma adriamycin levels were significantly higher between 4–12h following administration of adriamycin in niosomes, and this was reflected in a higher mean AUC. As it is impossible to take multiple plasma samples from single mice, it is not possible statistically to compare AUCs as this parameter is derived from single samples from multiple animals. Nevertheless, the form of the plasma concentration profiles differs. Adriamycin clearance (free solution = $0.51 l h^{-1}$; niosomes = $0.38 l h^{-1}$), volume of distribution (free solution = 5.7 l; niosomes = 1.1 l) and apparent terminal plasma half lives (free solution = 8 h; niosomes = 2 h) were calculated. The high volume of distribution indicates extensive tissue binding of adriamycin. It was not possible to detect a second phase of decline in plasma drug concentration for niosomal adriamycin, however, if plasma sampling had been continued for a further 12–24h biphasic decline in plasma levels may have become apparent.

Hepatic tissue levels of adriamycin were similar regardless of the formulation. The AUCs, peak hepatic levels (Table III) and issue half-lives (17h) for adriamycin were statistically indistinguishable. The degree of hepatic metabolism of drug was greater for niosome encapsulated adriamycin as found by Rogerson *et al.* (1987) in related experiments (Figure 4). Peak levels of the 7-deoxyaglycone of adriamycinol are significantly higher ($P<0.05$) after niosome treatment. The degree of hepatic metabolism can be defined by the ratio:

$$[\text{AUC adriamycin}]/[\text{AUC adriamycin} + \text{AUC metabolites}] \times 100\%$$

The metabolism ratio for niosomal adriamycin (85%) is less than for free adriamycin (95%) despite similar AUCs for parent drug, implying greater hepatic uptake and metabolism for adriamycin encapsulated in niosomes.

Peak renal levels are significantly ($P<0.05$) higher after free adriamycin. The renal AUC is also higher (Table III) and the tissue half life rather shorter (20.6h *vs.* 36.4h) than for niosomal adriamycin. There was a minor degree (<5%) of metabolism of adriamycin to the deoxyaglycones of parent drug and adriamycinol, which was similar for free and niosomal drug.

Cardiac adriamycin concentrations were significantly and consistently higher throughout the concentration time profile (Figure 5) in the free adriamycin group. Peak level, AUC and the terminal half life (36h *vs.* 10.8h) were elevated over those values observed with the niosomes (Table III). There was no significant intracardiac metabolism of adriamycin with either preparation of the drug.

Intratumoural drug levels were similar with regard to AUC (Table III). Peak concentration of adriamycin was higher following niosomal treatment ($p<0.05$). The terminal half life is apparently prolonged in mice treated with free adriamycin. However, this accounts for only a small fraction of the total AUC (<5%). No intratumoural metabolites were detected.

**Table III** Comparative total tissue and plasma contents of adriamycin following administration of free and niosome encapsulated drug. Peak levels are expressed at $\mu g g^{-1}$ ($\mu g ml^{-1}$ for plasma) ± 1 s.d. and the AUC as $\mu g g^{-1}$ tissue × hour ($\mu g ml^{-1}$.h for plasma).

| | *Free adriamycin* | | *Niosome encapsulated adriamycin* | |
|---|---|---|---|---|
| | *Peak level* ($\mu g ml^{-1}$) | *AUC* ($\mu g h ml^{-1}$) (0– ) | *Peak level* ($\mu g ml^{-1}$) | *AUC* ($\mu g h ml^{-1}$) (0– ) |
| Plasma | 0.1±0.01 | 0.4 | 0.075±0.01[a] | 0.53 |
| Heart | 11.7±0.10 | 110 | 5±0.27[a] | 43.6 |
| Kidney | 24±1.8 | 295 | 11.3±1.2[a] | 160 |
| Liver | 27.9±5.9 | 195 | 14.2±4.5 | 175 |
| Tumour | 4.7±0.6 | 30 | 6.9±0.9[a] | 41 |

[a]Indicates significant ($P<0.05$) differences in peak levels.

**Figure 4** Metabolites of adriamycin measured within the liver: 7-deoxyaglycone of adriamycin (●) 7-deoxyaglycone of adriamycinol (○) following administration of free solution (A) or in niosomal form (B). The vertical bars denote 1 s.d. of drug concentration.

### Discussion

The cytotoxic activity of adriamycin is maintained *in vitro* and *in vivo* despite encapsulation in niosomal vesicles. The niosomes possibly interact with cells *in vitro* in several different ways. Adsorption is likely to be a non-specific lipid-surfactant interaction occurring at the cell surface. This could allow locally high concentrations of drug to accumulate at the cell surface following drug diffusion out of the niosomes, thus promoting cellular uptake of drug. Alternatively, adsorption may lead to endocytic incorporation of the niosomes and their contents into the cell. Following endocytic uptake, the vesicles are presumably degraded within the cell thus allowing the drug to diffuse to its site of action within the nucleus.

Spheroid growth delay for niosomal adriamycin is virtually the same as for free adriamycin. Although soluble surfactants may increase cell permeability, it is difficult to

**Figure 5** Cardiac tissue levels of adriamycin following administration as free solution (●) or in niosomal form (○). The vertical bars denote 1 s.d. of drug concentration.
* = $P<0.05$, ** = $P<0.01$, comparing concentrations for free and niosomal adriamycin.

envisage non-ionic surfactant niosomes disrupting spheroid structure or diffusing intact into the spheroid, therefore presumably enough adriamycin is taken up by the external cell annulus to allow production of a concentration gradient sufficiently large to match the growth delay associated with free adriamycin.

Encapsulation of adriamycin in niosome vesicles alters the pharmacokinetics and disposition of the drug in tumour xenograft bearing nude mice. The significant differences, relative to administration of adriamycin solution, are as follows; prolonged release of drug from the plasma compartment with lower peak levels; lower peak cardiac levels of adriamycin with a shorter tissue half life and decreased cardiac AUC; a greater degree of hepatic metabolism to inactive 7-deoxyaglycones.

Cardiac levels of adriamycin are significantly lower with the niosomal drug preparation. Peak plasma levels of adriamycin are thought to be directly correlated with the development of cardiomyopathy and this concept underlay the use of prolonged infusion of adriamycin in chemotherapeutic regimens in order to decrease peak drug levels and hence the chance of cardiac damage (Unverferth *et al.*, 1982). The niosomes decreased peak plasma and cardiac adriamycin concentrations and total cardiac drug exposure (as assessed by the lowered tissue AUC). Although the cardiac toxicity of the niosomes relative to equimolar doses of adriamycin has not been formally assessed, it is possible, on pharmacokinetic grounds, that the niosomes will be relatively cardioprotective.

On the basis of tumour drug exposure (tumour adriamycin AUC), one would predict that free and niosome bound adriamycin would have similar cytotoxic efficacy. This appears to be the case, with similar tumour volume doubling times in the treated groups (Table II). In view of the finding of indistinguishable antitumour activity and the alluded association between intracardiac adriamycin levels and the subsequent development of cardiomyopathy, then niosome encapsulated adriamycin would seem to have a higher therapeutic ratio than free adriamycin.

Although the niosomes are chemically and physiochemically distinct from liposomes structurally they bear many similarities, and the results reported in this study are qualitatively similar to those in previously executed studies employing liposomes as the carrier vehicle for adriamycin (Gregoriadis, 1976; Kimelberg & Mayhew, 1978; Forssen & Tokes, 1983; Olsen *et al.*, 1982).

It can be concluded that niosome encapsulated adriamycin has a different tissue distribution from that of free adriamycin, with the predominant effect being lowered cardiac drug concentrations. It is possible, as total dose limitation in antineoplastic therapy is related to development of cardiomyopathy, that even a modest reduction in cardiotoxicity could enable an increase in the total dose of adriamycin administered for a reduction in the likelihood of toxicity.

The authors are grateful for the financial support of the Science and Engineering Research Council, to A.R., the Cancer Research Campaign and to L'Oreal, France, for the provision of surfactants and permission to publish this paper and to Mr T. Hamilton for expert technical assistance.

### References

AZMIN, M.N., FLORENCE, A.T., HANDJANI-VILA, R.M. STUART, J.F.B., VANLERBERGHE, G. & WHITTAKER, J.S. (1985). The effect of non-ionic surfactant vesicles (niosome) entrapment on the absorption and distribution of methotrexate in mice. *J. Pharm. Pharmacol.*, **37**, 237.

AZMIN, M.N., FLORENCE, A.T., HANDJANI-VILA, R.M., VANLERBERGHE, G. & WHITTAKER, J.S. (1986). The effect of niosomes and polysorbate 80 on the metabolism and excretion of methotrexate in the mouse. *J. Microencapsulation*, **3**, 95.

BAILLIE, A.J., FLORENCE, A.T., HUME, L.R., MUIRHEAD, G. & ROGERSON, A. (1985). The preparation and properties of niosomes–non-ionic surfactant vesicles. *J. Pharm. Pharmacol.*, **37**, 863.

CUMMINGS, J., STUART, J.F.B. & CALMAN, K.C. (1984). Determination of adriamycin, adriamycinol and their 7-deoxyaglycones in human serum by high performance liquid chromatography. *J. Chromatography*, **311**, 125.

DEPREZ-DE CAMPANEERE, D., BAURAIN, R., HUYBRECHTS, M. & TROUET, A. (1979). Comparative study in mice of the toxicity; pharmacology and the therapeutic activity of daunorubicin-DNA and doxorubicin-DNA complexes. *Cancer Chemother. Pharmacol.*, **2**, 25.

FLORENCE, A.T., BAILLIE, A.J., HALBERT, G.W. & WILLMOTT, N. (1988). Carrier systems for drug delivery and targeting: Protein microspheres, niosomes and LDL particles. In *The Influence of New Technology on Medical Practice*, Paul, J. (ed) p. 000. Macmillan: London (in press).

FORSSEN, E.A. & TOKES, Z.A. (1983). Improved therapeutic benefits of doxorubicin by entrapment in anionic liposomes. *Cancer Res.*, **43**, 546.

GABIZON, A., PERETZ, T., BEN-YOSEF, R., CATANE R., BIRAN, S. & BURENHOLZ, Y. (1986). Phase I study with liposome-associated and adriamycin: Preliminary report. *Proc. Am. Assoc. Cancer Res.*, **169**, 43.

GREGORIADIS, G. (1982). The carrier potential of liposomes in biology and medicine. *N. Engl. J. Med.*, **295**, 704.

KANEKO, Y. (1981). Thyrotropin–daunomycin conjugate shows receptor mediated cytotoxicity in cultures thyroid cells. *Horm. Metab. Res.*, **13**, 110.

KERR, D.J., WHELDON, T.E., KERR, A.M., FRESHNEY, R.I. & KAYE, S.B. (1986). The effect of adriamycin and 4′-deoxydoxorubicin on cell survival of human lung cells grown in monolayer and as spheroids. *Br. J. Cancer*, **54** (3), 423.

KIMELBERG, H.K. & MAYHEW, E. (1978). Properties and biological effects of liposomes and their uses in pharmacology and toxicology. *Crit. Rev. Pharmacol. Toxicol.*, **6**, 25.

MERRY, S., COURTNEY, E.R., FEATHERS, C.A., KAYE, S.B. & FRESHNEY, R.I. (1987). Circumvention of drug resistance in human non-small cell lung cancer *in vitro* by verapamil. *Br. J. Cancer*, **56**, 401.

MERRY, S., CUNNINGHAM, D., COURTNEY, E.R., HAMILTON, T., KAYE, S.B. & FRESHNEY, R.I. (1988). Circumvention of inherent resistance with verapamil in a human tumour xenograft. In *Human Tumour Xenografts in Anticancer Drug Development*, Winograd *et al.* (eds) ESO Monographs, p. 000. Springer-Verlag: Berlin, Heidelberg.

OLSEN, F., MAYHEW, E., MASLOW, D., RUSTUM, Y. & SZOKA, F. (1982). Characterisation, toxicity and therapeutic efficacy of adriamycin encapsulated in liposomes. *Eur. J. Cancer Clin. Oncol.*, **18**, 167.

POSTE, G. (1983). Liposome targetting *in vivo*; problems and opportunities. *Biol. Cell.*, **47**, 19.

RAHMAN, A., CARMICHAEL, D., HARRIS, M. & ROL, J.K. (1976). Comparative pharmacokinetics of free doxorubicin and doxorubicin entrapped in cardiolipin liposomes. *Cancer Res.*, **46**, 2205.

ROGERSON, A., CUMMINGS, J. & FLORENCE, A.T. (1987). Distribution of adriamycin in mice following administration of niosomes. *J. Pharm. Pharmaco.* (in press).

SETO, M., UMEMOTO, N. SAITO, M., MASUKO, Y., HARA, T. & TAKAHASHI, T. (1982). Monoclonal antibody anti-MM46: Ricin A chain conjugate: *In vitro* and *in vivo* antitumour activity. *Cancer Res.*, **42**, 5209.

SZEBERKE, M., WARE, R. & WHISON, M.E. (1972). The use of macromolecules as carriers of cytotoxic groups (part 2). Nitrogen mustard – protein complexes. *Neoplasma*, **19**, 211.

TWENTYMAN, P.R. (1982). Growth delay in small EMT6 spheroids induced by cytotoxic drugs and its modification by misonidazole pretreatment under hypoxic conditions. *Br. J. Cancer*, **45**, 565.

UNVERFERTH Dv., MAGORIEN, R.D., LEIER, C.V. & BALCERZAK, S.P. (1982). Doxorubicin cardiotoxicity. *Cancer Treat. Rev.*, **9**, 149.

YUHAS, J.M., LI, A.P., MARTINEZ, A.O. & LODMAN, A.J. (1977). A simplified method for production and growth of multicellular tumour spheroids. *Cancer Res.*, **37**, 3634.